# Mobilising communities for *Aedes aegypti* control: the SEPA approach

**DOI:** 10.1186/s12889-017-4298-4

**Published:** 2017-05-30

**Authors:** Robert J. Ledogar, Jorge Arosteguí, Carlos Hernández-Alvarez, Arcadio Morales-Perez, Elizabeth Nava-Aguilera, José Legorreta-Soberanis, Harold Suazo-Laguna, Alejandro Belli, Jorge Laucirica, Josefina Coloma, Eva Harris, Neil Andersson

**Affiliations:** 1 CIETinternational, New York, NY USA; 2 CIET in Nicaragua, Managua, Nicaragua; 3 Centro de Investigación de Enfermedades Tropicales (CIET), Universidad Autónoma de Guerrero, Acapulco, Mexico; 4 International Union against Tuberculosis and Lung Disease, Mexico City, Mexico; 5 Division of Infectious Diseases and Vaccinology, School of Public Health, University of California, Berkeley, Berkeley, CA USA; 6 Department of Family Medicine, McGill University, Montreal, Canada

**Keywords:** Sepa, Community, Community ownership, Dengue, *Aedes aegypti*, Complex interventions

## Abstract

**Abstract:**

Camino Verde (the Green Way) is an evidence-based community mobilisation tool for prevention of dengue and other mosquito-borne viral diseases. Its effectiveness was demonstrated in a cluster-randomised controlled trial conducted in 2010–2013 in Nicaragua and Mexico. The common approach that brought functional consistency to the Camino Verde intervention in both Mexico and Nicaragua is Socialisation of Evidence for Participatory Action (SEPA).

In this article, we explain the SEPA concept and its theoretical origins, giving examples of its previous application in different countries and contexts. We describe how the approach was used in the Camino Verde intervention, with details that show commonalities and differences in the application of the approach in Mexico and Nicaragua. We discuss issues of cost, replicability and sustainability, and comment on which components of the intervention were most important to its success. In complex interventions, multiple components act in synergy to produce change. Among key factors in the success of Camino Verde were the use of community volunteers called brigadistas, the house-to-house visits they conducted, the use of evidence derived from the communities themselves, and community ownership of the undertaking.

Communities received the intervention by random assignment; dengue was not necessarily their greatest concern. The very nature of the dengue threat dictated many of the actions that needed to be taken at household and neighbourhood levels to control it. But within these parameters, communities exercised a large degree of control over the intervention and displayed considerable ingenuity in the process.

**Trial registration:**

ISRCTN27581154.

## Background

In a 2011–13 cluster-randomised controlled trial in Managua, Nicaragua, and three coastal regions of Guerrero, Mexico, a pesticide-free, evidence-based approach to community mobilisation, called Camino Verde (Green Way), reduced dengue virus infection among children, self-reported dengue illness at all ages, and all dengue vector entomological indices [1].

Vertically organized and insecticide-based vector control efforts have important limitations, and there is a need to develop and test community-based strategies that include environmental management [2]. Up to the present, Camino Verde is the only randomised controlled trial to demonstrate impact of community mobilisation on dengue virus infection and reported dengue illness. However, a number of trials and other studies have reported the impact of community-based strategies on entomological indices [3–12].

These community-based strategies, used in various countries, all involve some form of community participation, and they are all aimed at controlling the mosquitoes that carry the virus, *Aedes aegypti*. Actions reported from trials and other studies include education of households through their children [4, 5, 7], adult education, distribution of printed recommendations and use of mass media [8], community workshops [4], provision of water container covers, clean-up campaigns, and involvement of women’s economic self-help groups [6]. Several involve engaging community members to act as mobilisers and educators [4–7].

Most community-based efforts have included, or were conducted in parallel with, the use of chemical larvicides and/or insecticide spraying, but others, called eco-friendly interventions, used only bio-control agents such as small fish and crustaceans that consume larvae in water receptacles or “ovitraps” that use non-chemical toxins to kill mosquito eggs [9–11].

Although all these interventions were community-based, many appear to have been driven for the most part from above. However, in four randomised controlled trials that were successful in reducing entomological indicators, two studies from Cuba and one each from Fortaleza, Brazil, and Chennai, India, there was serious community participation that helped tailor the interventions to local realities [3–6].

In this article, we describe how the Camino Verde trial incorporated several of the actions mentioned above, along with others, within a broader approach called SEPA (Socialising Evidence for Participatory Action). We describe the elements of the SEPA approach using the TIDieR reporting guidelines for describing interventions [13], discuss issues of cost, replicability and sustainability, and comment on which components of the intervention were most important to its success.

### The SEPA approach

Over 30 years, CIET – a non-governmental research organisation that grew out of the Centro de Investigación de Enfermedades Tropicales (CIET) at the Autonomous University of Guerrero in Mexico (http://www.ciet.org) – has developed an approach to the production and use of evidence for health promotion and community development, which we call “socialising evidence for participatory action” (SEPA).

Socialising evidence among stakeholders is more than just passing on research results. It begins with the research itself, partnering with communities to better identify and solve their development problems, using, wherever possible, participatory research designs and community engagement in various phases of the research process. These include open circulation, interpretation and collective discussion of local evidence, as well as the building of consensus on the choices for action. In this context, evidence is communicated, but not for prescribing a specific course of action. CIET socialises the evidence for people to seek solutions in dialogue with their own reality, in an informed manner but on their own terms, which often implies working out conflicting views and interests.

Unlike most health communication, SEPA does not seek individual behavioural change in and of itself but rather participatory action leading to change at individual, household, community, district, provincial and national levels, depending on the issues and the circumstances. Therefore, SEPA is better defined by its social components and its social and cultural implications than by individual perceptions and decisions.

#### Theoretical origins of the SEPA approach

The SEPA concept has its roots in a set of approaches variously known as action research, community research, participatory research, participatory action research, and participatory rural appraisal [14]. While there are long traditions of community research in North America, for the authors the intellectual leadership has come mainly from Latin America, Italy and the United Kingdom. The Latin American tradition of participatory action research (PAR) is rooted in the work of Paulo Freire [15], the Italian influence comes from their labour movement’s alternativa operaia [16] and the United Kingdom influence from Robert Chambers [17]. Common threads in these philosophies are community ownership of both the information and the research process, the premise that research will lead to action for the benefit of the community, and the weaving of research into a process of community reflection and learning. PAR has a fairly long history in education, organisational development, and rural development and has more recently been incorporated into health research, most frequently under the name of community-based participatory research (CBPR). An influential 2004 report found that a frequent drawback of CBPR and related approaches was the lack of generalisability; results were often difficult to apply beyond the group of participants involved in a given study [18, 19]. Since then, the number and quality of CBPR studies has mushroomed [20, 21]. Although some have found that evidence of impact and outcomes attributable to it continues to be scarce [22, 23], it appears that CBPR is particularly effective in addressing health disparities [20, 24–26] and in resolving problems related to survey and intervention design [27–29].

Unlike Freire and CBPR, where the community itself sets the research agenda and may even maintain control over the outcomes of the research [30, 31], SEPA usually operates within a framework where agendas are set by the providers of research funding. This sets up a tension between donor-driven research and community-led research. The resolution we have tried to apply is that, whatever the limitations imposed by the funding opportunity, SEPA can support generation of community-led solutions.

A guiding principle of the SEPA approach is that the results of community research should be able to withstand rigorous scientific scrutiny. The research and the potential actions it leads to are intended to have an influence beyond the places where they are conducted, and their impact must be measured. The backbone of the method is epidemiological, and the use of evidence is crucial. Evidence plays a fundamental role in SEPA as a tool for rational persuasion [32, 33]. In a community context, evidence can stimulate reflection and dialogue, leading to new collective interpretations and consensus for action. Just as people tend to be more open to evidence when they see its subject as something that affects their own situation, their responsiveness increases when this evidence is actionable [34, 35].

SEPA differs from social marketing, social advocacy and social mobilisation. Certain social marketing tools, including mass media, may be used at both the intervention and dissemination stages of SEPA, but they are not inherently part of the process. Some elements of social mobilisation are present in SEPA, so far as it implies dialogue and action at the level of government, public services and communities, and between these spheres. But SEPA mobilisation seeks to avoid the pitfalls of social manipulation. Rather, it is a way of stimulating dialogue that seeks to strengthen collective awareness and interest around the issues and the evidence, thus hopefully contributing to an increasingly informed, self-sustained environment for participatory action and change.

The notion of community participation in primary health care was given strong impetus at the 1978 Alma Ata conference on Health for All, when its participants declared that primary health care “requires and promotes maximum community and individual self-reliance and participation in the planning, organisation, operation and control of primary health care....” [36]. In the ensuing years, this key principle of primary health care has been more observed in the breach than in reality [37–39], but it has remained a guiding principle of SEPA.

#### **Previous experience with SEPA**

Previous SEPA experiences supported the development of the Camino Verde intervention. SEPA was at the core of a micro-regional planning initiative in Mexico from 1992 to 1995. Micro-regional planning translated local epidemiological research results through participatory analysis into information suitable for communication and local action planning [40]. In Nicaragua since 1998, CIET has conducted six social audits on community perception of corruption in, and satisfaction with, public services; the presentation of the results as the authentic voice of the community to public authorities has resulted in significant changes in public policy [41]. Recent and current CIET social audits incorporate cluster-randomised controlled trials (CRCTs) to test alternative ways of achieving social objectives [42]. A CRCT in a social audit context explored the effect of evidence-based training of front-line health workers for health promotion in the Sindh province of Pakistan [43]; another CRCT in Pakistan tested a low-cost community-based approach to extending the coverage of childhood vaccination in the Lasbela district. This intervention doubled the odds of measles vaccination in the intervention communities and trebled the odds in favour of full DPT vaccination [44]. A current CRCT in Botswana tests the impact on HIV incidence of a combined package of structural interventions focused on choice-disabled young women [45].

While in the Camino Verde trial, SEPA operated almost entirely at the community level, in other instances the approach has been used at the level of planners and policy makers through workshops around evidence presented in the form of summary findings, maps and score cards, not to prescribe solutions but to assist in the interpretation of evidence [41, 46, 47].

## **How SEPA was applied in the Camino Verde trial**

### Actors

A variety of actors played key roles in implementing the SEPA strategy in the Camino Verde trial.

#### The brigadistas

These mobilisers and educators constituted the backbone of the effort. All were residents of the communities where they conducted SEPA activities and all had to be acceptable to other community members. Facilitators (see below) trained them in the life cycle and habits of the dengue virus-transmitting *Aedes aegypti* mosquito and the dengue virus transmission cycle. Brigadistas’ training included accompanying facilitators in making initial contact with households. Volunteers who joined brigades after the initial contacts were usually trained by other brigadistas.

#### The facilitators

The facilitators’ role was to (1) make initial contact with the community and facilitate a brigadista recruitment process, (2) present evidence from the baseline survey, (3) provide training, and (4) support the community in its assuming of responsibility for the intervention. In Nicaragua, the facilitators were former brigadistas active in the 2004–2008 feasibility study on the same subject. During this pilot experience, the research team came to appreciate the sense of solidarity that makes daily life possible in the neighbourhoods of Managua and to understand that its role was to reinforce values of respect for individual differences and collective responsibility already present in the community and pass this appreciation on to the first generation of brigadistas in the trial. Facilitators in Mexico, mostly recent graduates from the University of Guerrero where CIET is located, received more formal ethical training in which a Mexican communications expert and a member of the Nicaraguan field team participated. In both countries, facilitators sought to move as quickly as possible from leadership roles to supporting roles.

#### The households

Environmental control of the *Aedes aegypti* mosquito at the household level was indispensable to the entire effort. All consenting households in the research clusters participated in the intervention, and all members of each household were invited to join in the effort. While the clusters used to measure the effects of the intervention were limited to approximately 140 households, the intervention often reached households in the surrounding neighbourhood as well.

#### Community leaders

The Nicaraguan trial was entirely concentrated in the capital city, Managua, where neighbourhoods typically have recognized, active leadership closely allied with the Sandinista government. The SEPA strategy there was to work with these leaders and deliberately avoid creating parallel structures, while striving to maintain the brigade’s autonomy and political neutrality. Several brigadistas were also community leaders. The Mexican trial covered the entire coastal area of the state of Guerrero. In Guerrero’s rural areas, the strategy was similar to that in Nicaragua, especially where the communities are primarily indigenous and more organised. In urban areas, mainly in the city of Acapulco, identifiable community leadership tended to be less unified and less effective for our purposes. The organisation of many urban communities has been disrupted by drug-related violence, and the Camino Verde brigades in some cases helped to restore community organization.

#### Children

Children of both sexes were very active in the intervention – in their own households, in the schools, and in collective neighbourhood activities. Some brigades in Nicaragua were made up predominantly of children. In Mexico, two student brigadistas were selected to coordinate activities in each of the schools. Once made aware of what mosquito larvae and pupae look like up-close, children can become fascinated with them and become dedicated foot-soldiers in the struggle to eliminate them.

#### Other organisations

Numerous national and regional organisations in both countries, while usually not rooted in any individual community, are active at the grassroots level. These organisations had diverse main agendas, but the threat of dengue and the need for mosquito control was a common concern. The SEPA programme partnered with as many of these organisations as possible in its mobilisation activities.

### Activities

A feasibility study in Nicaragua between 2004 and 2008 [48] developed six main strategic components:

- the use of community volunteers, called brigadistas;
- house-to-house visits, called *visitas de acompañamiento*;
- simple mosquito control tools accessible to every household;
- collective elimination of breeding sites not under the control of individual households;
- engaging schools, churches, shops, clubs and other organizations in the effort;
- a wide variety of media used to educate and motivate.

The first three of these activities went together in the household visit. The initial interaction with residents leading to an invitation to enter the home was a critical moment in the relationship between brigades and community members, as it raised key issues of consent, trust, and confidentiality. Once *brigadistas* were allowed inside the dwelling, the dialogue about dengue was engaged and reinforced by joint inspection of water receptacles and discussion about how to prevent infestation with mosquito larvae and pupae.

Since, however, dengue is not just a household problem but also a community one, brigadistas were called upon to encourage collective action to eliminate the vector from abandoned properties, public spaces such as cemeteries, parks, playing fields, bus stations, and central squares, and private commercial spaces. School children, churchgoers and club members were often willing participants in these collective actions and they were also active in their own homes reinforcing the lessons imparted by the household visits. Media for educating and mobilising community members included graffiti, murals, banners, street theatre, parades, pamphlets, piñatas, festivals and songs.

To launch the intervention, facilitators convened and ran intervention design groups to discuss survey results, cost implications, and specific prevention strategies in each community. In Nicaragua, these groups were composed of community leaders and were focused on evidence about costs of dengue illness and money spent on personal protection against mosquitoes. Each participant was given a sheet of paper with four questions, each question accompanied by the evidence from the baseline survey specific to her/his neighbourhood. Each question was discussed by the group and the group formulated group responses or commentaries [49]. In Mexico, where neighbourhood leadership was less homogeneous, design groups in each neighbourhood consisted of whoever had some knowledge of the community – district leaders or anyone else who could contribute to developing strategies for reaching and informing the community members. They discussed results from the baseline survey and were asked to identify actions that could be taken at the household level to control the mosquito and identify ways for brigadistas to disseminate the information throughout the communities.

Details, including photographs of these activities can be found at: http://caminoverde.ciet.org/en/nicaragua/activities/and http://caminoverde.ciet.org/en/mexico/activities/

### Materials

The principal material used in Camino Verde was evidence. The evidence was biological, entomological, epidemiological, and economic. The biological evidence concerns the development cycle of the immature *Aedes aegypti* mosquito. The entomological evidence was of two kinds: a) aggregate numbers and percentages of mosquito larvae and pupae derived from systematic inspections of household and community water receptacles and b) visual demonstration of the presence of these larvae and pupae to householders on their own premises. The epidemiological evidence came from the baseline study of the Camino Verde trial in the same communities and concerned risks from failure to protect against dengue and likelihoods of protection from various actions that households and communities could take to minimize those risks. The economic evidence was a) cost data on dengue and dengue control gathered in the baseline surveys and b) reflection by each household on the costs they incur from seeking treatment for dengue illness and from purchases of anti-mosquito chemicals and devices.

**Fig. 1:**
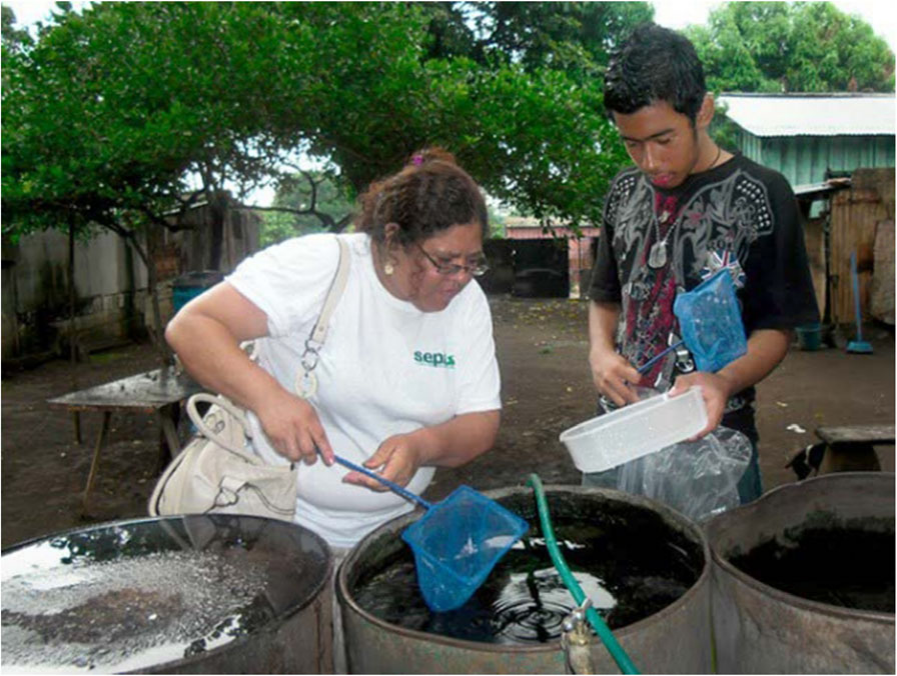
Brigadista demonstrating to a householder in Nicaragua the presence of larvae in his own water storage barrel. Photo credit: Alejandro Belli

One piece of evidence was lurking in each household’s own water containers. During the household visits, brigadistas would accompany the household head, and often the children, on an inspection of the various water receptacles on their property. Finding mosquito larvae and/or pupae right on the premises offered a unique opportunity for making people aware of their presence and discussing ways of preventing them from maturing and/or from ever being there in the first place (Fig. 1). Rather than prescribe ways of doing this, brigadistas discussed various solutions with residents and encouraged them to be creative in seeking solutions. It was out of such dialogues that residents in Mexico brought up the tradition of using fish for biological control of mosquito larvae [50]. In Nicaragua, the idea of producing elasticized barrel covers emerged from such discussions. Thus, evidence from the baseline survey was used to launch the intervention.

**Fig. 2:**
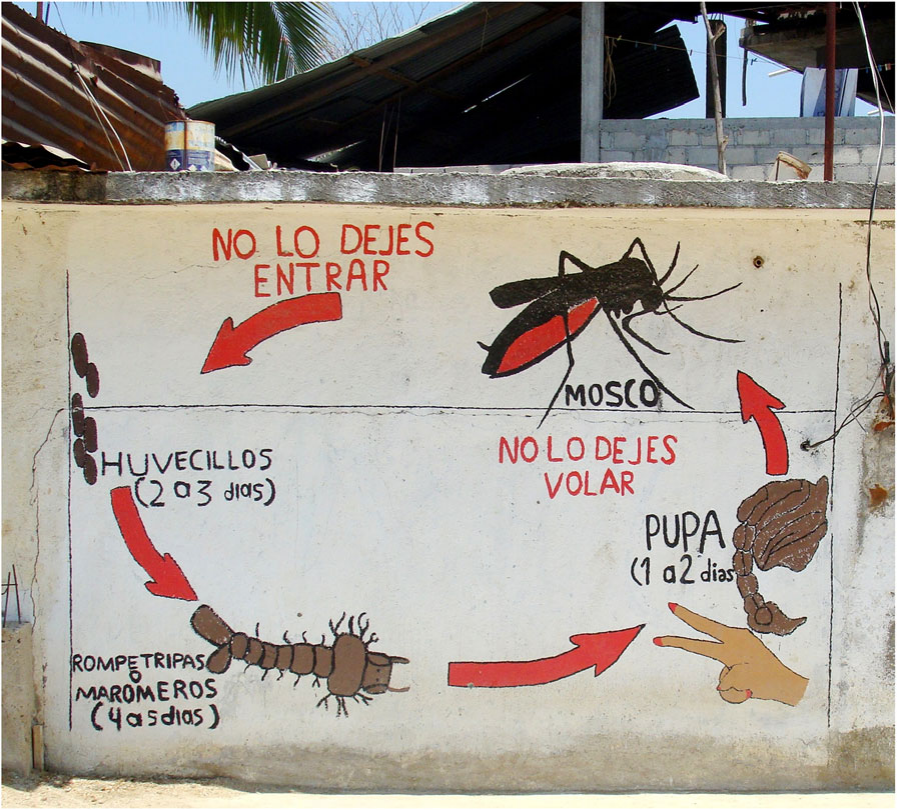
Community mural from Mexico depicting the *Aedes aegypti* development cycle. Photo credit: E.A. Undurraga, PLOS NTD 10.1371/journal.pntd.0003547

At the household level, brigadistas carried a water jar or plastic bag containing live mosquito larvae and simple tools (a strainer, flashlight, magnifying glass, plastic pan and plastic pipette) for collecting larvae in the household. A particularly important tool was a laminated graphic showing the mosquito vector’s life cycle on one side, with alternative control measures and the advantages and disadvantages of each option on the other. This graphic was also displayed on the t-shirts worn by many of the brigadistas, who used it while explaining to households how to interrupt the cycle before the mosquito matures. The graphic was also projected or otherwise displayed during collective events and included in murals and posters in public places (Fig. 2).

At the neighbourhood level, brigadistas helped organize various collective events – both awareness-raising events and events to control mosquito breeding in public spaces. Materials used for awareness-raising events included leaflets, posters, parades, songs, games, murals, graffiti, t-shirts and piñatas. Examples of these events and the materials used can be found at http://caminoverde.ciet.org/en/nicaragua/activities/ and http://caminoverde.ciet.org/en/mexico/activities/and also at http://caminoverde.ciet.org/en/galleries/photos/.

### Modes of delivery

At the household level, brigadistas delivered the intervention face to face. At the community level, brigadistas and facilitators delivered the intervention both in small groups and at large public events. The only electronic tool used was a blog created in Nicaragua (http://sepa-nic.blogspot.com) where community *brigadistas*, residents and leaders could give testimonies of their own experiences, share anecdotes, enjoy photos, access their own data and work tools, monitor the product of their own work, and ask, comment and learn about what was happening in other neighbourhoods [51].

### Location

The Camino Verde intervention took place in the city of Managua, Nicaragua, and in three coastal regions of the state of Guerrero in Mexico. Maps showing the relative locations are presented in the main article on the trial [1]. The Nicaraguan population involved was entirely urban, while in Mexico it was both urban and rural. In Mexico, most of the awareness-raising events like parades, murals, etc. took place in the urban areas (Acapulco), whereas biological control of mosquito larvae using fish occurred mainly in the rural areas. Mexican facilitators working outside Acapulco were each responsible for 3 communities. They travelled by public transport and often found lodging in or near one of their communities before proceeding to the others, as travel by night was not safe.

### Time frame

Figure 2 of the main article summarises the timeline of the trial [1]. It shows the intervention itself taking place from August 2011 to November 2012. Before field work could be started, however, there was a great deal of preparatory work to be done. SEPA needs to be introduced with respect for the different rhythms at which individuals, communities and public institutions operate. The need to foster a favourable environment is common sense to all those with any experience in community mobilisation, but the focus on evidence requires a special non-didactic stance on the part of researchers who, with limited time and resources, may be tempted to prescribe ways of achieving common objectives and thus weaken individual and community autonomy. The time and resources required to inform and to engage communities, establishing a climate of dialogue and mutual trust, can be costly, but if community leaders do not perceive that they have a real voice in the undertaking and a responsibility for its outcome, their commitment to the effort is likely to be half-hearted at best.

The intervention was preceded by baseline surveys during which saliva samples from children aged 3 to 9 were collected at the beginning and end of the peak dengue season (September to December 2010) in order to measure recent dengue virus infection. The baseline also gathered demographic and socio-economic data, data on dengue illness and its costs to the household, on knowledge about dengue and its vectors, attitudes and practices related to dengue control, and expenditures on anti-mosquito products. Results from the saliva samples were returned to the individual households in both intervention and control communities. Meanwhile, we contacted local health authorities, local NGOs, and community leaders to obtain their consent and cooperation. Finally, before launching the intervention, we held a series of community discussions of baseline results. In Nicaragua, discussions with community leaders focused on evidence about costs to households from dengue illness and money spent on personal protection against mosquitoes [49]. In Mexico, focus groups in each neighbourhood discussed these and other results from the baseline survey.

## **Adapting the intervention and fidelity to the intervention design**

Hawe and colleagues have argued that, in complex interventions, the function and process of the intervention should be standardized, rather than the components themselves, thus allowing the form to be tailored to local conditions [52]. We designed the Camino Verde intervention from this perspective. In this case, the process standardised was SEPA, the sharing (socialisation) of evidence with community residents and leaders in ways that elicit household and community action to prevent the spread of dengue virus.

This difference between function and form is apparent at different levels. At the inter-country level, it can be seen in the difference between Mexico and Nicaragua in the way that brigadistas were recruited, trained and rewarded. In Nicaragua, all the brigadistas were volunteers who responded to an invitation from community leaders. There was a good deal of turnover among the brigadistas, but as some left the brigades, there was a steady stream of replacements who were trained by those remaining. Some brigadistas eventually took on broader roles, joining the leadership of their already-existing neighbourhood structures but with an identity that was free of past conflicts [51]. In Mexico, brigadistas were community members selected during initial focus group meetings or appointed by community leaders who were trained by facilitators and received a modest financial stimulus (about USD 90 per month), although other community members, especially children, joined the brigades voluntarily.

Another difference between countries was introduction of biological control using fish, practiced in Mexico but not in Nicaragua. Brigadistas and facilitators in Mexico learned that in some intervention communities, residents kept fish in water containers, using them to prevent the development of mosquito larvae. They then encouraged other communities to do the same. Secondary analysis of the Camino Verde results in Mexico provides evidence that fish in water containers can reduce the risk of dengue virus infection and dengue illness [50].

At the neighbourhood level, some examples of how communities interfaced with public services show how useless it would have been to arrive with preconceived solutions. In one Mexican community, members decided to conduct a street cleaning campaign. They first had to obtain the cooperation of the sanitation services. Having had success in doing this they went further and arranged for monthly clean-ups with the cooperation of that department plus those of water supply and public works. In Nicaragua, what citizens learned from accompanying the entomological inspections in their own homes led to the realization that full control of the mosquito problem required community-wide solutions. This led to collective action to put pressure on small businesses and repair shops to control infestation on their premises, and this in turn gave them confidence to deal with public institutions such as the local health centre and the municipal authorities responsible for refuse collection and the repair and maintenance of streets and drains.

In another Mexican community, the lack of regular garbage collection had led one resident to dump her garbage in empty lots and even sometimes on property belonging to other residents. This became the cause of chronic tension among the residents, who requested help from the brigades in resolving it. They first visited the woman and with her consent arranged a meeting between her and her neighbours, the upshot of which was a petition to the municipal sanitation services for increased garbage collection in the whole community.

Appendix 2 to the main Camino Verde report presents lists of commonalities and differences in the way that SEPA was implemented in Mexico and Nicaragua. There were aspects common to both countries by reason of the trial’s content and objectives, aspects common to both countries by reason of the experience gained in the feasibility study conducted in Nicaragua before the trial, and aspects that differed by country and/or community [1].

Fidelity to the SEPA approach was assured by supervision and by exchanges among communities. Both countries made use of peer monitoring. Brigadistas from one community visited another community under supervision of a different facilitator without intervention on the part of researchers. They applied a brief questionnaire and conducted entomological inspections in consenting households. Afterwards, the visiting brigadistas presented their findings to the host brigadistas and together they discussed possible adjustments to the approaches being used. Meetings among facilitators and brigadistas from different communities also helped to assure commonality of purpose and process.

## **Discussion**

We now know that the Camino Verde trial was successful in reducing dengue virus infection among children, self-reported dengue illness at all ages, and all dengue vector entomological indices in Managua, Nicaragua, and three coastal regions of Guerrero, Mexico. SEPA is not a recipe. It is an approach that serves a common function of assisting communities – always with respect for their knowledge and customs – to identify ways of reaching common objectives. Nevertheless, if we want the Camino Verde approach to be scaled-up and replicated elsewhere, we need to identify some practical aspects that appear to be essential to success.

### What worked?

Studies of community-based dengue control strategies have been criticized for failing to distinguish which specific components of the intervention have the greatest impact on *Aedes aegypti* control [8]. Guidance on process evaluation of complex interventions from the UK Medical Research Council, on the other hand, has pointed out that complex interventions are by definition intended to be greater than the sum of their parts, with multiple components acting in synergy to produce change, and that attempts to understand parts of the intervention should always be considered in relation to the functioning of the intervention as a whole [53]. An approach that encourages communities to develop their own solutions to the problem, essentially the key to success, makes it even more difficult to measure the impact of specific components.

The two most consistent strategic elements in the Camino Verde trial were the use of community volunteers (brigadistas) and repeated house-to-house visits (visitas de acompañamiento).

Brigadistas, a variety of community health workers [54], were the front line of the intervention. Key qualities required of a brigadista were: to be a member of the community in which they worked, to be acceptable to community leaders and members, to be able to gain access to households so as to talk to residents and accompany them on an inspection of the water receptacles on the property, and to be able to work together with fellow brigadistas on collective activities to eliminate breeding sites not under the control of individual households.

The house-to-house visits were the brigadistas’ most regular activity, weekly in principle but frequent at least. The method most consistently used was the demonstration to householders of the presence of mosquito larvae and pupae on their own premises. In Mexico, an anthropologist from the University of Guerrero analysed householders’ narratives of change collected during the trial and summarized his findings in these terms:

1. What appears to be a key aspect of this process is “learning by doing” and the “ownership effect” (efecto de lo propio) … in which learning about the mosquito’s reproductive cycle, the recognition of larvae, pupae and eggs, and learning what to do about it (how to clean [the receptacle]… how to cover it, etc.) is accompanied by inspection and sometimes by actual cleaning….
2. The repetition of the visits makes possible a transition from distrust to confidence and also reinforces what people have learned. Posters and street theatre then act as complementary reinforcement of what has been learned.
3. The attitude of the brigadistas and their motivation also encourages residents to maintain their surroundings properly.

--From a 2011 internal report by Prof. Joan Muela.

Thus, the brigadistas (with their specific qualities) and the household visits appear to be the most “active ingredients” responsible for the trial’s success, but underlying the brigadistas’ role and their household visits was also the climate of community trust and community ownership of the process.

Randomised controlled trials in Fortaleza, Brazil [4] and Chennai, India [6] that were effective in reducing entomological indices did not use the equivalent of brigadistas. Nicaragua has had brigadistas working on the dengue problem well before Camino Verde [55]. Mexico too had its “block activators” (activadores de manzana), persons in the community who educate their neighbours in ways to identify and control mosquito breeding sites [56]. However, these people were generally not effective in preventing dengue. Although they came from the community, they were answerable more to the health authorities than to their communities.

As long ago as 1951, Lewin argued that the process of ‘unfreezing’ existing behaviour patterns needs to take place in a group environment and to involve open and supportive communication among those involved in negotiating the change [57]. And, in the context of environmental activism, Jackson has argued that changing behaviour cannot be conceived as the processes of encouraging change at the individual level; rather, behavioural change has to be a social process [58].

From this perspective, it is unlikely that the presence of brigadistas and their household visits alone account for the success of Camino Verde. Although there is no direct proof for it, we believe that community ownership of the undertaking was also a key factor and that attempts to replicate the Camino Verde trial’s success while discounting the importance of community autonomy and community control of the social change process are unlikely to succeed [59]. The WHO Study Group definition of community health workers states that they should be members of the communities where they work, selected by the communities and answerable to the communities for their activities [60]. Interventions involving community health workers under these criteria for malaria control, health education, breastfeeding promotion, newborn care and mother psycho-social well-being have proven their effectiveness [61].

### **Sustainability**

Secondary results of the trial show behavioural differences between households in the intervention and control communities. In the trial’s follow-up survey, the household respondents in intervention communities reported higher levels of collective self-efficacy: agreeing that communities themselves can do something to control mosquitoes in their environment ([1]; Table 3 in this reference).

The field team in Nicaragua took specific measures to promote sustainability. From the start, brigadistas were volunteers, and responsibility for incidental costs was deliberately passed from the facilitators to the community leadership. Community leaders were actively involved, some even joining the brigades. And, by maintaining close relations with Ministry of Health officials, keeping them informed and inviting them to Camino Verde events, the team created a promising climate of openness on the part of the authorities to further trials of the approach [51]. Unpublished data from a survey conducted in Managua in 2015 offered indications that households exposed to the 2011–2012 intervention were more likely to say they check for mosquito larvae when they do patio cleaning and to associate brushing or scrubbing barrel walls with elimination of mosquito eggs, indicating that these households retained knowledge and attitudes acquired during the intervention.

In Mexico, there is evidence that the use of larvivorous fish in water storage containers has spread beyond the original trial communities through word of mouth (A. Morales, personal communication, 12/25/2015).

While sustainability ultimately depends on the will and capacity of community members to control their own environment, some external stimulus and guidance will be needed. During the trial, the external actors were the facilitators whose work came to an end once the trial ended. In Nicaragua, where communities and government work together in many endeavours, we have reason to expect that government will step in and make the Camino Verde process its own, at least on a trial basis. In some parts of Mexico, where community trust in the government is weaker, non-governmental solutions may be required.

### Costs

Creating favourable environments and fostering community ownership of the SEPA process are time-consuming activities and are not inexpensive. We have collaborated with health economists on an as yet unpublished economic analysis of the trial outcomes. Preliminary results show that the annualized cost of the intervention per member of the intervention community in Mexico was USD 16.72 while that for Nicaragua was USD 7.47. Using the perspective of the World Health Organization’s “Choosing Interventions that are Cost-Effective” project [62], the intervention, as conducted during the trial, was found to be too costly to be considered cost-effective.

This cost-effectiveness analysis excludes two significant economic aspects of the Camino Verde intervention. One is the savings from dengue cases averted, including the number of work and/or school days that patients and caregivers might have lost but were saved thanks to the intervention [63]. The other aspect concerns what economists call “externalities” such as expanded community leadership, conflict resolution, voluntarism and growing confidence in the communities to demand services which, though difficult to quantify, are of real and lasting benefit.

Nevertheless, our efforts going forward will include achieving more favourable cost-effectiveness, which could be done in three ways: by achieving the same result at a lower cost; by improving the result for the same cost; and/or by achieving more than one objective at a comparable cost. First, as we had no precedent for measuring the impact of community mobilisation on dengue virus infection incidence and reported dengue illness, we were less concerned about costs than ensuring a favourable result, and we spent the entire budget provided by the sponsor to achieve it. We believe that all three ways of improving cost-effectiveness will apply here. First, with the experience now acquired, we believe that economies of scale and other measures could make the intervention more efficient. Secondly, we have evidence to suggest that the intervention could be made more effective for a similar cost. Analysis of the trial’s intermediate outcomes from a gender perspective, presented in a companion article, shows that what the trial achieved was due almost entirely to the women in the intervention communities [64]. We need to test ways of involving males more fully in the process. If we can achieve this, there is potential for a much greater impact [59]. Thirdly, the *Aedes aegypti* mosquito is a vector not only for dengue but also for chikungunya, Zika, yellow fever and other diseases. We do not have proof of Camino Verde’s impact on these diseases, but it is likely to be comparable with the impact on dengue. For example, the prevention of *Aedes aegypti* proliferation could make Camino Verde cost-effective for its effect on Zika alone. Given the risk of microcephaly from Zika, the resulting benefits would be substantial [65–67].

We also know that the communities themselves did not always consider dengue control to be their highest priority [49, 50]. Broadening the portfolio of the *brigadistas* to deal with other health conditions would not only respond to community priorities, but would also be another way to achieve additional objectives for comparable costs. The house-to-house visits and community events that were part of Camino Verde could well become the occasions for educating and encouraging households and communities toward other preventive actions such as immunisation, screening for other infectious and chronic diseases, and healthier behaviours.

For these reasons, therefore, we believe that the SEPA approach can be judged cost-effective, building on lessons learned from the Camino Verde trial.

### Limitations

It would be foolish to pretend, nonetheless, that a self-sustaining culture of vigilance and cooperation in response to the *Aedes aegypti* threat has been permanently embedded in any of the communities that participated in the Camino Verde trial. The real gains achieved in this trial need to be consolidated and extended until a societal tipping point is reached where it will be normal for all households to monitor water receptacles on their own properties and cooperate with their neighbours and public and private institutions in a shared responsibility for thorough and consistent control of their environment to make it free of dengue, chikungunya, Zika, and other arboviruses. We should not exaggerate the degree of autonomy exercised by the communities in this trial. They did not choose dengue as the subject they most wanted to mobilise around and they received the intervention by random assignment. The very nature of the dengue threat dictated many of the actions that needed to be taken to control it.

However, within these limitations, communities exercised a large degree of control and displayed considerable interest and ingenuity in the process. None of the investigators in Mexico, for example, had thought of biological control before it was suggested by community members. In Nicaragua, the researchers played no direct role in the selection of *brigadistas.* Community leaders invited neighbours to join the brigades, and many leaders themselves joined the brigades. And in both countries, communities were empowered by evidence to interact with public authorities in new and creative ways.

## Conclusion

The SEPA approach is an appropriate one for mobilising communities to combat diseases transmitted by the *Aedes aegypti* mosquito and is applicable in different community and country conditions.

## Abbreviations

CBPR: Community-based participatory research
CRCT: Cluster-randomised controlled trial
PAR: Participatory action research
SEPA: Socialisation of evidence for participatory action (In Spanish, *sepa* is the imperative form of the verb *saber* – to know)
TIDieR: Template for intervention description and replication
